# Moment Approximation of Infection Dynamics in a Population of Moving Hosts

**DOI:** 10.1371/journal.pone.0051760

**Published:** 2012-12-18

**Authors:** Bruno Bonté, Jean-Denis Mathias, Raphaël Duboz

**Affiliations:** 1 Laboratory of Engineering for Complex System (LISC) of the French National Research Institute for Science and Techniques in Environment and Agriculture (IRSTEA), Aubière, France; 2 Animals and Integrated Risk Management (AGIRs) research unit of the French Center for International Cooperation for Agricultural Research and Development (CIRAD), Montpellier, France; 3 Computer Science and Information Management (CSIM) department of the Asian Institute of Technology (AIT), Pathumthani, Thailand; Cinvestav-Merida, Mexico

## Abstract

The modelling of contact processes between hosts is of key importance in epidemiology. Current studies have mainly focused on networks with stationary structures, although we know these structures to be dynamic with continuous appearance and disappearance of links over time. In the case of moving individuals, the contact network cannot be established. Individual-based models (IBMs) can simulate the individual behaviours involved in the contact process. However, with very large populations, they can be hard to simulate and study due to the computational costs. We use the moment approximation (MA) method to approximate a stochastic IBM with an aggregated deterministic model. We illustrate the method with an application in animal epidemiology: the spread of the highly pathogenic virus H5N1 of avian influenza in a poultry flock. The MA method is explained in a didactic way so that it can be reused and extended. We compare the simulation results of three models: 1. an IBM, 2. a MA, and 3. a mean-field (MF). The results show a close agreement between the MA model and the IBM. They highlight the importance for the models to capture the displacement behaviours and the contact processes in the study of disease spread. We also illustrate an original way of using different models of the same system to learn more about the system itself, and about the representation we build of it.

## Introduction

The structure of the contact network of individuals plays an important role in the dynamic spread of infectious diseases. Different contact structures lead to different dynamics of infection [1], [2]. We know that mean field models, which hypothesize a constant homogeneous contact rate between susceptible individuals and infectious individuals [3], can not always be taken as an acceptable approximation. Clusters of infected individuals appear during epidemics, implying that the infection rate can exhibit complex spatio-temporal dynamics [4], [5], which depend on the structure of the contact network [6]. For particular situations and scales, this structure should thus be taken into account [1], [7], [8].

For these reasons, many recent models consider the spread of infectious diseases as a diffusion process within a contact network [9], [10], raising the issue of how the contact network can be drawn. In some cases, the network is built from population surveys at the appropriate scale (see [11] for an example in human and [12] in animal). However, the contact network is difficult to design. In most cases, the full population cannot be sampled and network building relies on descriptive statistics. Using these statistics, some models can also approximate the impact of the network structure on the disease spread [6], [13]. Unfortunately, these statistics themselves are not always available. This is often the case in animal health: the protocols used in ecology to build these statistics are not always able to produce reliable statistics for epidemiology [14]. Accordingly, a current challenge in the field of disease spread modelling is accurately reproducing the infection dynamics at the population level, from minimum information on the structure of the contact network between individuals. To tackle this issue, individual movements can be simulated to generate potential contact patterns between individuals. Individual-based models (IBMs) have been built for disease transmission [15]–[17]. IBMs are mechanistic and often stochastic models that meet several difficulties regarding their rigorous communication [18] and their sensitivity analysis and parameter estimation [19]. In addition, IBMs can not be analytically analysed at the population scale to study asymptotic behaviours.

The motivation of this work is to provide a mathematically tractable model which takes into accounts the dynamic contact structure of a moving hosts population. We use a moment approximation [20], [21] of an IBM. This method has already been used to model disease spread in networks where the structure of contacts is known [8], [22], [23]. Here, we approximate an IBM that explicitly simulates the individual movements. In this way, we take into account the dynamics of a contact process that does not involve any contact network. The aim of this work is to test wether such an approximation is valid and to discuss the impact of individual movements on epidemic dynamics. To this end, we compare the simulation results of three models: 1. an IBM with moving hosts, 2. a moment approximation of the IBM (called MA), 3. a mean field approximation of the IBM (called MF). The MA is specific to this IBM and simulates up to its second moment. The MF is generic and usually used in epidemiology, it simulates only the first moment of the IBM dynamic.

We choose the example of the highly pathogenic avian influenza virus (HPAIV) H5N1 spreading in a commercial poultry flock. HPAIV H5N1 is still of great public concern today owing to the risk of zoonosis [24]. Theoretically, a transmissible infectious disease may persist within a network of epidemic units [2]. For HPAIV H5N1, surveillance and control of the disease must be considered at regional or national scales considering a network in which epidemic units are poultry flocks. From this point of view, experience in Thailand showed that different poultry production types play different roles in sustaining transmission [25]: backyard poultry flocks have a different impact from commercial poultry flocks. In this context, we consider that it is important to provide new simulation models for these different poultry production types. These models must (i) accurately simulate disease spread dynamics, and (ii) not incur a too-high computation cost, so that they can be included in wider meta-population models representing the epidemics at national or regional scales. Here, we focus on commercial poultry flocks. We consider a flock of broiler chickens where poultry are free to move about in a closed environment like a shed. This kind of system is easier to model than backyard poultry flocks because it is composed of a fixed, homogeneous population (a mono-specific cohort) maintained in a closed space. It is an interesting example of an epidemic system with moving individuals that can be simulated with a limited number of rules. It is important to accurately capture the dynamics of disease spread in such poultry production flocks, because outbreaks in commercial poultry have been found to be disproportionately infectious [25].

## Individual-based Model

### 1. Description of the Model Approaches

We consider a population of *N* individuals. Each individual is identified by a unique number *i* such that 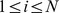, and has two attributes: its position in two-dimensional space (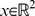) and an epidemiological state (noted 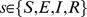). Epidemiological state separates the host population into four sub-populations of individuals: (i) susceptible individuals (*S*), which are disease-free but can become infected when exposed to the virus, (ii) exposed individuals (*E*), which have been infected but are not yet themselves infectious during a so called latency period (they harbour the multiplying virus but do not shed it), (iii) infectious individuals (*I*), which shed the virus and can transmit the infection to susceptible individuals, and (iv) removed individuals (*R*), which have been infected and have either been killed by the disease or have recovered from infection and are now permanently immunized. Note that the removed individuals are still accounted for in the population. We use formal notations to describe the IBM. Position and epidemiological states of individual *i* at time *t* are respectively noted 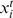 and 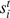. We assume that we simulate a representative area of the space and that we have a fixed population in a closed space. For this reason we use a toroidal space. This assumption fits the scenario of a disease spreading in a poultry flock in which no individual enters or leaves the population.

Although we have chosen the example of HPAIV H5N1, we only consider direct transmission. Indirect transmission has been many times discussed for this disease (including transmission through water). A recent study in complex artificial aquatic biotopes shows that persistence of infectious HPAIV do not exceed 4 days in rain water, and that there is no evidence of such infectious virus in mud samples even if viral RNA can be found [26]. Even if the environment is still considered as being a potential reservoir, we assume here it has a negligible effect when compared with direct transmission during an outbreak.

The model dynamics comprises four processes: (i) individual movement, (ii) infection of susceptible individuals by infectious individuals, (iii) an incubation process for exposed individuals whereby they become infectious and start shedding the virus, and (iv) recovery or death of infectious individuals.

### 2. Individual Movement

The same movement process is used for all individuals (*S*, *E* and *I*). We assume that each individual moves following a kernel function 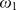. The kernel function can be Gaussian or simply a local window. In our case, the kernel is a local uniform kernel. The instantaneous probability that during an infinitesimal time 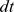, an individual moves to some location *x* from its previous location 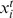 is given by the function 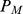 with:
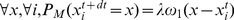
(1)


The movement rate 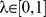 represents the probability that an individual moves during *dt*.

### 3. Infection Process

This is the process whereby a susceptible individual becomes exposed due to infectious contact with infectious individuals. We assume that the expected contact rate between two individuals *i* and *j* is given by a normalized kernel function 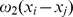 multiplied by a basic contact rate *k*. Hence we consider that a contact structure exists instantaneously: at any time, any individual *i* is linked to any individual *j* by a valued link with the value 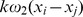. We also assume that each contact with an infectious individual has a probability *c* of exposing it to the virus. 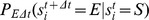 is the probability that the susceptible individual *i* becomes infected during 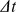. We can then compute 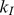, the expected contact rate with infectious individuals as:
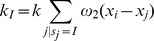
and the expected number of contacts with infectious individuals during 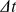 is 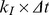. The probability that a susceptible individual *i* is not infected during 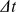 is then [27]:



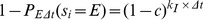
(2)We then compute the instantaneous probability of infection 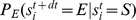 as the limit of Equation 2 when 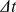 tends to zero. If we note 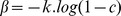, we obtain the following expression for 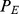 (Equation 3):
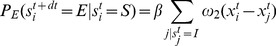
(3)


The constant 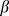 is called the infection rate. This limit computation, and the whole hypothesis and principles leading to Equation 3, are quite usual in epidemiology (details can be found in [27] chapter 2 box 2.1.). We detail them here because we want to describe how the contact structure is computed through the distances between individuals, and how it drives the infection process.

### 4. Incubation Process

The incubation process (virus multiplication inside a host) is characterized by the fact that at the end of the latency period, an exposed individual *i* (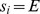) becomes infectious (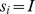). Hence we only represent the event leading to the change in the individual epidemiological state. The instantaneous probability that this event occurs, noted 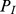, is constant over the latency period, as shown by Equation 4:
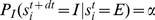
(4)where 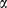 is called the incubation rate.

### 5. Removal Process

The instantaneous probability 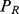 that an infectious individual *i* (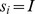) becomes removed (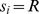) is given by Equation 5:
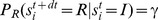
(5)where 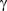 is the recovery rate parameter.

## Moment Approximation

We use a moment approximation closed at the third moment. The first moment is a statistic on a “mean” individual (here its expected infectious state), the second moment is a statistic on pairs of individuals, and the third moment is a statistic on triplets of individuals. The first moment is the expected mean densities of *S*, *E*, *I* and *R* individuals in the space (see Section 1). The second moment is the distribution of the distances between pairs of different types of individuals (see Section 2). The third moment is the distribution of triplet configurations of different types of individuals (see Appendix B.1 in Appendix S1). The first and the second moment are simulated considering all of the processes described in the IBM and the third moment is approximated using a combination of the values of the first and second moments.

### 1. Mean Densities of the Infectious States

We note 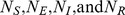 the mean densities of individuals *S*, *E*, *I* and *R* in the population. The dynamics of 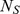 is specified in Equation 6

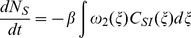
(6)where 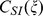 is the expected “susceptible individuals - infectious individuals” pair (*SI*-pair) density with locations situated at a vector 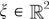 apart. For a given set of individuals and a given configuration 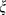, the pair density is defined as:
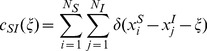
(7)with 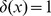 if 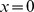 and 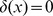 otherwise. 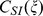 is the mathematical expected value of the function 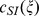 regarding the possible distributions of the individuals and their probabilities (A formal definition and an account of the utility of the 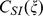 pattern summary can be found in [20]).

For *E* and *I* individuals, mean dynamics are respectively specified by Equations 8 and 9:
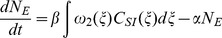
(8)

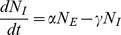
(9)


Finally, as we consider a static density of individuals *N*, the density of removed individuals can be simply computed as follows:
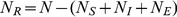
(10)


The dynamics of mean densities of infectious states depend on the mean local density of infectious individuals around the susceptible individuals. This mean local density is traduced by the pair correlation densitiy 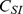 which appears in the infection terms of Equations 6 and 8. Its dynamics is developed in the next section.

### 2. Mean Densities of the Pair Correlations

**Figure 1:**
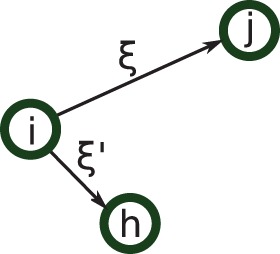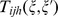 triplet configuration. Individuals *i* and *j* are separated by a distance 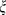, and individuals *i* and *h* by a distance 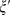.

In this section we explain how we built the equation of the 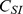 dynamics from the IBM mathematical specification (see Equation 11). Other necessary pair correlation dynamics, as well as triplet dynamics, are described in appendices (see Appendix B in Appendix S1)
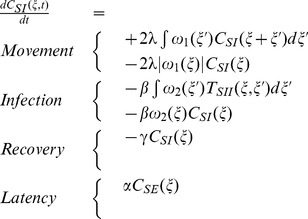
(11)where for all 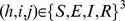, 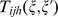 represents the correlation density of triplets as shown in Figure 1. 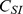 dynamics depends on the four processes presented in the above equation:

- The movement terms can be split into two terms. The first one computes the expected number of new pairs. A pair at distance 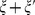 is considered here. When an individual of this pair moves to a distance 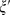, a new pair is created with a distance 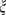. The second term computes the expected number of destroyed pairs. When an individual of an *SI*-pair at distance 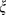 moves, it destroys the pair. These terms are multiplied by 2 because we are considering the potential movement of both individuals.
- The infection terms correspond to the exposure to virus of the individual 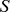 of the *SI*-pair. There are two possibilities. The first one depends on the triplet configuration between a susceptible and two infectious individuals (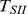) and on the interaction kernel 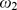. When the *S* individual of a *SI*-pair is infected by another infectious individual located at distance 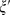, it destroys the pair. The second possibility lies in a too-short distance of the *I* and *S* individuals of a *SI*-pair, following the interaction kernel 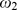. In this case, the *SI*-pair is destroyed by the infection of the susceptible individual.
- In the recovery term, we consider the number of infectious individuals in situation 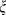 with respect to a susceptible individual. This individual has a probability 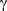 of recovering and thus destroying a *SI*-pair.
- The latency term corresponds to the number of exposed individuals that are in a configuration at a distance 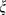 with a *S* individual and hence create a new *SI*-pair at a distance 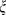 if they become *I*.

The description of the 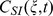 dynamics is important for an understanding of the different mechanisms that construct the contact network; it let us view the contact network indirectly. Interestingly, it depends on the 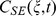 dynamics and on the triplet configuration 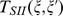.

### Mean Field Approximation

In the first moment approximation, we consider that the spatial structure is uniform at any time. In this case, we can express the second moment as a function of the first moments: 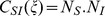. Information about the spatial structure is lost. In this case, the equations of the first moments are simplified:
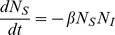
(12)

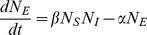
(13)

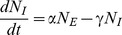
(14)


It is noteworthy that this system is the same as the most classic mean field model used in epidemiology, usually called the SEIR compartmental model. It has been widely developed and is still of considerable interest [28]. In this model, the number of individuals is stationary and the space is not represented. The force of infection 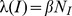 is termed density-dependent because it depends only on the infectious individual mean density 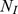.

For the same reasons as explained in the “Individual Based Model” Section (Subsection 3), we can write the instantaneous infection probability for any susceptible individual as follows (as shown in [[27], box 2.1]):
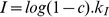
(15)where 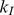 is the expected contact rate with infectious individuals and *c* is the probability of being infected if a contact occurs. Equation 12 is the computation of the expected number of new infectious individuals during *dt* and can be written as:



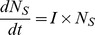
(16)Hence when we use Equation 12, we assume that for each susceptible individual,
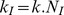
where *k* in units of 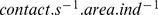 is a constant contact rate per unit of infectious individual density. In other words, using the mean field model, we assume that the contact structure is a complete graph in which all link values are equal (the value is: 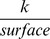).

## Experimental Design

### 1. Objective

The objective is to compare the simulation results of the IBM, MF and MA. Comparison between IBM and MF results tells us whether the spatial pattern plays a role in the dynamics. We have seen in the model presentations that MF considers a homogeneous spatial structure and that the MA simulates the contact process and a part of the spatial structure dynamics. Conversely, the IBM is our reference and simulates the dynamics of the entire spatial structure. Thus if MA results are closer than MF results to IBM results, we can assume that MA successfully incorporates spatial pattern dynamics.

For each parameters values combination, we simulate the MF, the MA and the IBM. For the IBM, five simulations are run each time and only the mean result is reported. The model discretization used for implementation is presented in Appendix A in Appendix S1. The grid definition is equal to [150×150]. A convergence study was conducted on the grid size, and this value is sufficient to obtain adequate discretization.

The initial configuration represents an epidemic situation in which several individuals are already infectious when the simulation starts. In our example of HPAIV, it represents a case where an infected breeder supplied the flock and consequently a part of the population is infected. The initial state consists in 9000 individuals *S* and 1000 individuals *I* which are randomly distributed in the space. Due to these initial conditions, variability is low for the IBM because we have many individuals and several infected individuals at the beginning of the simulation. Thus, even if we have only five replicates for each set of parameter values, the differences between the aggregated models (MA and MF) and the IBM are not due to the stochastic property of the IBM.

### 2. Parameters Values

We identify possible intervals for the values of each parameter (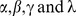) and we test four values taken with a regular step in each interval: intervals of parameters 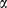 and 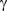 are estimated from laboratory experiments results published in [29]; intervals of paramters 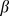 and 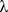 are calibrated using field data published in [30], [31]. Table 1 summaries the intervals and the sources used to build the intervals.

**Table 1:** Parameters values.

par	name	unit	interval	sources
*α*	incubation rate	min^−1^	[1/360, 1]	[29]
*β*	infection rate	min^−1^.ind^−1^	[0.001, 0.1]	[30], [31]
*γ*	recovery rate	min^−1^	[1/2880, 1/720]	[29]
*λ*	mobility rate	min^−1^	[0.1, 1]	[30], [31]

#### 2.1. Estimation of 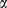-and 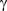-parameters

For 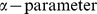 we choose a lower boundary corresponding to the mean latency period (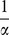) observed for this virus (corresponding to a 6-hour period [29]), and an upper boundary corresponding to no latency period. For parameter 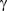, we choose an interval corresponding to the minimum and maximum infectious periods (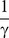) observed during laboratory experiments for this virus [29].

#### 2.2. Calibration of 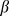-and 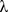-parameters


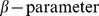
 incorporates many characteristics of the system under study and so there is no single value of 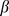 that depends only on the H5N1 virus. Such transmission parameters are difficult to estimate and are usually calibrated to fit prevalence data observed on the field. In the same way, the mobility characterized by parameter 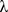 has not been evaluated in poultry flocks. For this reason, we use the IBM to calibrate the intervals for these parameters. We simulate outbreaks by changing initial conditions to one infectious individual and 9999 susceptible individuals: we want to make sure that the intervals chosen for 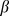 and 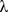 keep us within a realistic situation for H5N1 outbreaks observed on the field in poultry flocks [30], [31]. We choose intervals that keep us within situations where we observe from 90% mortality in 6 days (worst cases observed in [30], [31]) to situations where no epidemic starts.

### 3. Comparison Criterion

The criterion used to compare simulation results is defined according to our application example and to the dynamic features of the system. It is based on the first moment dynamics because we assume that it is determinant to consider the interaction of the poultry flock with a wider system. The infectious potential of a poultry flock depends on the density of exposed and infectious individuals in the poultry flock. Also, control measures require outbreak detection, which depends on the proportion of animals showing symptoms of the disease (infectious state), and of dead animals (removed state), but does not depend on animals incubating the virus (exposed state). Thus what is important to estimate is not so much the time lag after which the outbreak detection occurs, as the proportion of individuals exposed to the virus when the detection occurs.

Ideally, we would like to compare trajectories of the first moments, namely of the variables 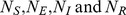. However, for the low values of the infectious rate 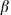, trajectories of IBM replicates can be significantly different. Indeed, the epidemics start more or less quickly due to the stochastic nature of the model. Except for this time shift, the replicates exhibit very similar results because many individuals are considered (10000 individuals). Consequently, we use a criterion which is independent of time and takes directly into account the relative densities of the different infectious states. The criterion is the function 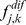 (where 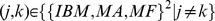), which compares the integrands of the dynamics. It is defined by Equation 17:
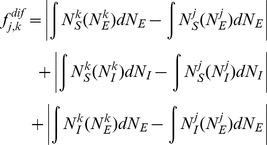
(17)


We note that the dynamics of 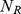 is not considered, in order to have a criterion based on three independent variables (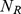 can be deduced from the other dynamics and is redundant). The value of 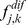 is calculated until the number of exposed individuals and the number of infectious individuals are both equal to 0 (i.e. when the system is considered as stationary).

## Results

### 1. Influence of the Parameters on the Spatial Structure

**Figure 2:**
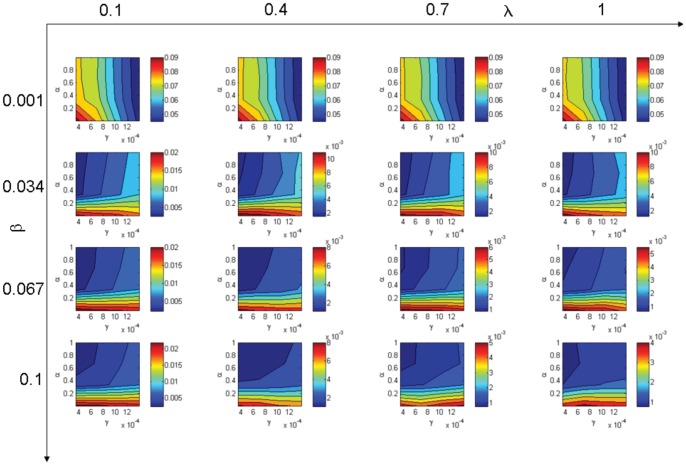
Difference between the IBM and the mean field (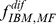). 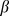
 is the infection rate, 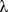 is the mobility rate, 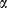 is the incubation rate and 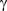 is the recovery rate. Hot colours stand for higher difference than cold colours.

Values obtained for the difference criteria 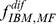 are presented in Figure 2. These values characterize the difference between the IBM results and the MF model results for the tested parameters. We recall that the IBM considers the whole spatial structure of the population and its dynamics, and that the MF model does not consider any spatial structure. Consequently, these results tell us about the influence of the parameter values on the impact that the spatial structure of the population has on the disease spread dynamics. Comments on these results follow:

- Influence of the latency (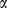) and the infection (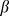): these parameters control the transition of the state of an individual from *S* to *I*. For high values of 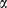 and 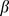, a susceptible individual rapidly becomes an infectious individual. This leads to clusters of infectious individuals. Before discussing the observed results, we explain what usually happens with static hosts. When individuals do not move, spatial autocorrelation of *I* individuals reduces the numbers of infectious contacts, because infectious individuals are surrounded by other infectious individuals and cannot reach susceptible individuals. Thus, increasing the values of parameters 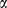 and 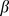 parameters intensifies this process and increases the distance between individual-based models and mean field models. Here, when individuals move, the interactions are more complex. For low values of 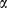 and 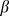, the difference between IBM and MF are higher than for high values. We note that in our model a high correlation density of *II*-pairs does not entail a low correlation density of *SI*-pairs. The determinant relation in the infection dynamics is the density of *SI*-pairs, which drives the potential infectious contacts. The MA presented in the next section describes the dynamics of these pair densities and gives a better prediction of the IBM dynamics. In Section 2 we illustrate the differences observed between the three models regarding the density of *SI*-pair correlations of individuals observed in the IBM simulations.
- For 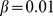, the difference is very high. This is because the disease spread does not begin in several simulations of the IBM owing to the low value of 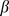.
- Influence of 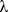: this parameter has a marked influence on the spatial structure: for high values of 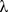, the mobility is high and the population distribution is very similar to a uniform distribution. In this case, it decreases the difference between IBM and MF; it is noticeable on the first column for 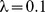, where the difference is considerable.
- Influence of 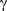: classical values of 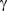 are low. This leads to a weak influence of 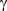 except in the case of low values of 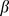 (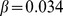) and high values of 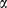 (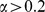). In this case, low values of 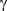 lead to lower differences. This is the expected trend, considering that a longer infectious period (small value of 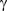) decreases the effect on spatial structure. We note that when mobility increases, the relative effect of 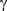 decreases. This is because all spatial structure influence is decreased by increasing the mobility.

The above comments apply only to the tested values of the parameters (see Table 1).

### 2. Comparison of the Moment Approximation with the IBM

**Figure 3:**
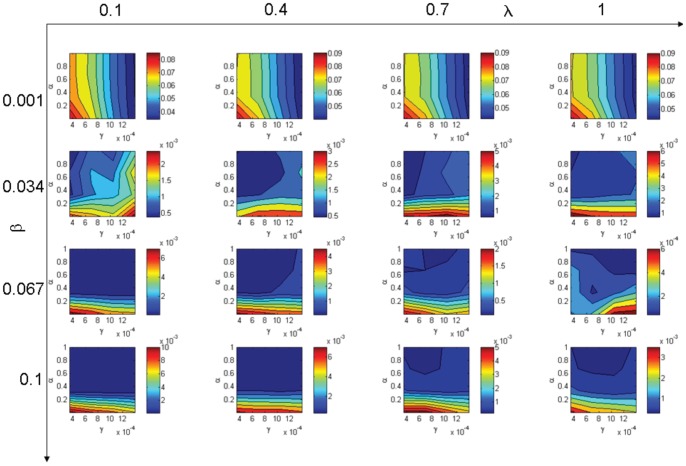
Difference between the IBM and the moment approximation (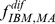). 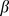
 is the infection rate, 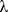 is the mobility rate, 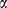 is the incubation rate and 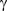 is the recovery rate. Hot colours stand for higher difference than cold colours.

Values obtained for the difference criteria 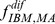 are presented in Figure 3. These values characterize the difference between the IBM results and the MA model for the tested parameters. From these results we can study the ability of the MA to reproduce the IBM results:

- The distribution of 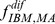 is qualitatively very similar to the distribution of 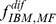. This means that the parameter values for which the MF model least successfully reproduces the IBM results are also those for which the MA model least successfully reproduces them. This can be explained by the fact that the MA simulates the spatial structure dynamics, but when the spatial structure becomes somehow too complex, the moment approximation does not fully capture the spatial structure: we have a second moment approximation, and it is not sufficient in the case of complex spatial structures.
- The difference between the IBM and the MA is less than the difference between the IBM and the MF. The moment approximation gives a better approximation of the dynamics than the mean field approximation because the MA takes into account the spatial structures.
- The influence of the movement rate 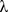 is the same as for 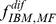. The similarity of the results increases with the rate of movement.

**Figure 4:**
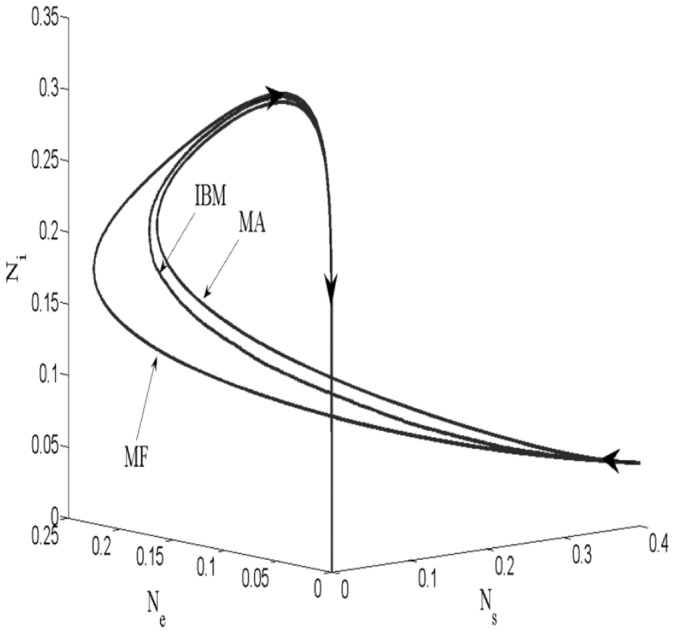
Plane of phases of the IBM (averaged over five replicates), the MA and the MF simulations. 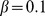
, 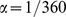, 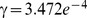 and 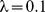.

**Figure 5:**
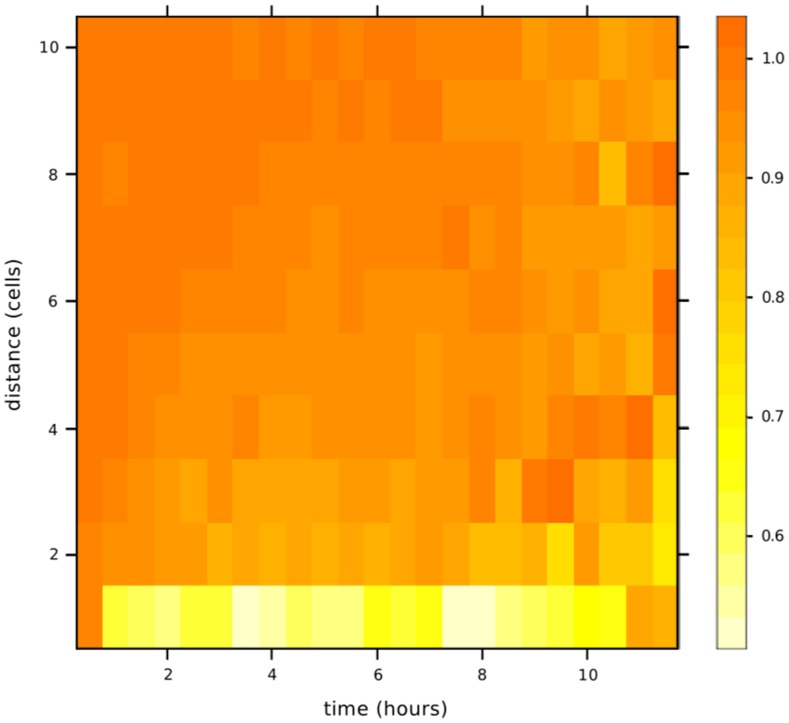
Dynamics of the normalized *SI*-pair correlation densities in the IBM for simulation of Figure 4. Pair correlation density (colour code) is computed for distances from 1 cell to 10 cells each simulated half-hour (30 time steps) until there are 10 susceptible individuals left (12 hours here).

To compare the three approaches, dynamics were plotted on Figure 4 in the case of a spatial structure where the difference between the MA and the IBM is high. We can note that the MA is very close to the IBM results and that the MF is not sufficient to approximate the IBM. We note that this figure shows the case where the difference between the IBM and the MA is the highest. In other cases, the dynamics of the IBM and the MA are superimposed. For this case (same parameters and initial conditions), we chose a simulation of the IBM and plotted the *SI*-pair correlation density evolution during the simulation (see Figure 5). Densities are normalized. This means that a density of 1 for a given distance implies that the density of pairs at this distance is the expected density if the population is homogeneously distributed. We can use the evolution of the *SI*-pairs correlation densities of individuals in the IBM (Figure 5) to discuss phase trajectories (Figure 4). Figure 5 shows that the average distance between *S* individuals and *I* individuals is increased at some time during the simulation. We can observe a clear pattern for the column that corresponds to the 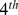 hour, for instance: the smallest densities are observed for the smallest distances. This means that a spatial structure appeared during the simulation. This spatial structure is responsible for the difference observed between the curves in Figure 4. The longer average distance between infectious and susceptible individuals leads to a smaller force of infection. This effect cannot be reproduced by the mean field model, and so the MF overestimates the number of *I* individuals. We note that when the simulation goes further, the spatial structure tends toward a uniform distribution.

## Discussion and Conclusion

We compared the simulations of the three models IBM, MA and MF for different values of parameters chosen through a regular exploration of the parameter space. As we develop it in the model presentation, the MF model does not consider any spatial structure. The MF approximation corresponds to a moment closure at the second moment, thus the same probability of contact is considered for any pair of individuals. Conversely, in the IBM, the probability of contact for a pair of individuals depends on the distance between the individuals, which in turn depends on the system history. Hence the comparison between IBM results and MF results give us a measurement of the influence of spatial structure on the disease spread dynamics for different values of the parameters. Doing this, we note that for the situation considered, the influence of spatial structure is not trivial and can not be summarized as the effect of spatial autocorrelation of infectious individuals.

In the MA, a part of the spatial structure is taken into account through the simulation of the second moment (dynamics of pair correlations densities). We observe that the simulation results of the MA are closer than the MF results to the IBM results. We also observe that in regard to our comparison criterion, MA results and IBM results are very close, and so we conclude that MA correctly integrates the dynamic spatial structure, even when mobility is low. Note that this model only considers direct transmission. As a consequence, the model applicability is limited to environmental conditions where the indirect transmission of HPAIV (through faeces or feathers for instance) is negligible.

As an application, we used the MA model to simulate HPAIV spread in a commercial poultry flock. Our comparison criterion takes into account the evolution of the relative proportions of the different states in the population. These relative proportions can be considered to evaluate the impact of surveillance and control measures: they enable to establish a relation between the detection process (linked to the proportion of *I* and *R* individuals) and the infection process (linked to the proportion of *I* and *E* individuals). We showed that especially for some of the parameter values, the MF model was unable to reproduce the IBM behaviour perfectly and that we obtained better results with the MA model than with the MF model. One of our ultimate aims was to produce a model that could be included in a wider model representing several poultry production units connected together. This study is a first step towards this goal, and shows that the MA model can be a good candidate over its range of validity. However, it is not stochastic, and so simulation results represent expected situations and do not enable us to discuss rare events. Also, the MA model considers mean values and so it is not relevant for small population sizes. Consequently, an epidemic starting with a single infected individual cannot be modelled with the MA model. Here, we chose to consider situations where we have 10% prevalence when the epidemics starts. In this way we studied the MA model within its validity domain. We note that this validity domain limits its use within a wider model, because an accidental infection of the flock, in which a single individual would be infected, cannot be considered. The same issue should be considered for the MF model, which also considers mean values. In response, it is possible to associate an IBM and a MA model. This was done in [32], where a hybrid epidemic model is built, changing from individual-based to mean field equation-based model during a simulation, depending on the size of the infectious population to be simulated, which evolves in the course of the simulation. Using such methods it is possible to use stochastic IBMs for epidemic start or specific perturbations and MA models for other situations.

We note that for each specific application, the comparison between IBM and MA models must be studied, and we would need to define a specific criterion that would enable us to discriminate when the MF model is sufficient, when the MA is necessary and sufficient, and when only an IBM is able to describe the dynamics. Such a criterion should be defined according to the modelling objectives. It can be a qualitative output, which tells us, for instance, wether the disease will become endemic in the population or wether it will vanish after an epidemic. This kind of behaviour can be observed, for instance, in the case of models with reintroductions of susceptible individuals. The system can fall into an equilibrium state where the number of infectious individuals never recedes to zero.

More theoretically, this first study shows that in cases where we cannot depict the contact network, the MA method does provide a compromise between the simulation of every individual movement and the simulation of a MF model, which would ignore any structure in the population.

Lack of information on contacts between animals has already led modellers to build IBMs based on animal behaviours provided by ethologists. They then use the outputs of the IBM to calibrate a metamodel that reproduces IBM dynamics. We define a metamodel (a model of a model) as a mathematical function that approximatively simulate the model response, has a negligible computation cost, and can accurately predict new responses [33]. A complete applied example of such a study is presented in [16] and [34]. In these papers, the authors first presented an IBM at the herd level [16], and then used a metamodel to simulate the herd level model in a wider model [34]. The IBM they use is more complex than ours, but is based on the same processes (rules for movement, contact, infection and recovery). The difference between these studies and the method we used here is that in our case we build the approximated model based on the processes modelled and not based on the outputs obtained, as is the case when modellers calibrate metamodels with model outputs. We can see in our discussion that the differences between the models and the approximated models can be explained by the level of complexity considered by the approximation.

With this study, we emphasize the advantages of using the MA method versus metamodelling methods based on calibration. We can see here that the MA method forces the modeller to focus on the processes driving the system. In our case, it gives the modeller an explicit formulation of contact dynamics. We consider that in those cases where such approximation is possible, models obtained with MA should be preferred to calibrated metamodels because of their transparency feature. Here, our approach to building the MA was to transfer the individual rules to pair-wise rules, and then to triplet-wise rules. In future work, it would be of interest to assess whether the same approach can be used for other existing IBMs with moving individuals that are used in epidemiology. We consider that such approach would be successful insofar as the IBM is clearly specified. However, tests remain to be done to assess what kind of individual rules (such as complex logical rules) are compatible with MA method and what kind in not compatible because too complex.

We may add that a deterministic model such as a MA model can be complementary to an IBM model, which is stochastic. In the context of epidemiological models, a stochastic model can be used to apprehend the uncertainty of the output of a decision when a deterministic model is used to understand and discuss determinants of a complex dynamics.

Apart from improving understanding and precision, we consider that simulating some features of the spatial patterns is valuable. These features are in our case the distance distribution between pairs of different types of individuals. It has been shown that these features can be used to evaluate models [35], and thus to better calibrate it. Moreover, new methods are developed to infer epidemics dynamics from distance distribution between pairs of infectious individuals in the case of static individuals [36]. We consider that if we can simulate the second moment successfully we will be able to use the same methods in the case of moving individuals.

To conclude, this study highlights the fact that MA techniques are not the prerogative of networks-based models: this technique can be useful in the case of infection dynamics in a population of moving hosts.

## Supporting Information

Appendix S1 (PDF)
