# Supplementary material for: Moment Approximation of Infection Dynamics in a Population of Moving Hosts
Source: PLoS One. 2012 Dec 18;7(12):e51760. doi: 10.1371/journal.pone.0051760 (PMC3525645; doi:10.1371/journal.pone.0051760)
Supplement: Appendix S1 — (PDF) [file pone.0051760.s001.pdf]

Appendix A IBM discretization and implementation

A.1 Discretization

To simulate the IBM, we consider a regular time step Δt and a discrete 2D-space (a square toroidal 8-neighbourhood lattice). We discretized the spatial kernels ω_1 and ω_2 for the movement and infection processes (Equations 1 and 3 of the paper). We used a uniform kernel function with a size equal to one discrete space step for the infection kernel ω_2 (See equation 1). This leads us to consider a contact per time step between two individuals if these individuals are located in a same cell during Δt:

$$\begin{aligned} \omega_2([0,0]) &= 1 \\ \omega_2(x \neq [0,0]) &= 0 \end{aligned} \tag{1}$$

We used a uniform kernel function with a size equal to three discrete space steps for the move kernel ω_1 (see Equation 2). The individual can only move in a neighbour square:

$$\begin{aligned} &\omega_1([x,y]) = \frac{1}{8} \text{ if } x = \pm 1 \text{ or } y = \pm 1 \\ &\omega_1([x,y]) = 0 \text{ otherwise} \end{aligned} \tag{2}$$

A.2 Initialization and dynamics of the discrete model

At the initialization, a state $\{x_i^0, s_i^0\}$ (with $x_i \in \mathbb{N}^2$ and $s_i \in \{S, E, I, R\}$) is given for each individual $i \in [1, N]$. Then, for each time step, for each individual, we compute the next state $(\{x_i^{t+\Delta t}, s_i^{t+\Delta t}\})$ according to the previous global state $(\{x_i^t, s_i^t\})_{1 \leq i \leq N}$.

- **Computing new infectious state $s_i^{t+\Delta t}$**
  According to wether the individual infectious state is respectively S, E or I, we perform a random selection using probability P_E, P_I or P_R computed with Equations 3, 4 or 5 of the paper to compute wether infectious state will be changed to respectively E, I or R. If the test succeeds, the state is changed, if not, it stays the same.

- **Computing new position $x_i^{t+\Delta t}$**
  We first compute wether an individual moves or stays in the same cell. We perform a random selection of probability $P = P_M(x_i^{t+\Delta t} \neq x_i^t)$. If the random selection fails, the position is not changed, if it succeeds, the new position is computed as $x_i^{t+\Delta t} = x_i^t + (x, y)$ with *(x,y)* randomly chosen in $\{(x, y) \mid x = \pm 1 \text{ and } y = \pm 1\}$

A.3 Computer implementation

The discrete model was implemented as a discrete event dynamic system with regular time step using the virtual laboratory environment (VLE) [1]. The VLE is a set of tools based on discrete event system Specification (DEVS) and application programming interfaces in C++ for modelling and simulation. VLE provides a random number generator, is interfaced with the R statistical software [2] and enables us to distribute the simulations of an experimental plan on several processors. We used the R statistical software to generate the experimental plans and analyse simulation results.

Appendix B Development and discretization of the moment approximation

Here, we present the development of pair correlation dynamics. The pair correlation $C_{SS}(\xi, t)$ can be calculated for the susceptible-susceptible pairs as follows:

$$\begin{aligned}
&\frac{dC_{SS}(\xi,t)}{dt} = \\
&Movement \begin{cases} 2\lambda \displaystyle\int \omega_1(\xi')C_{SS}(\xi+\xi')d\xi' \\ -2\lambda|\omega_1(\xi)|C_{SS}(\xi) \end{cases} \\
&Infection \Big\{ -2\beta \displaystyle\int \omega_2(\xi')T_{SSI}(\xi,\xi')d\xi'
\end{aligned} \tag{3}$$

The susceptible-susceptible pair correlation dynamics depends on the two processes in the above equation:

- The movement terms (the first two) are computed in the same way as for the C_{SI} pairs (see Subsection 2 in the "Moment Approximation" Section of the paper).
- The infection term computes the expected number of susceptible individuals in situation ξ that become infected. It depends on the triplet configuration T_{SSI} and the interaction kernel ω_2. If an infectious individual is located at distance ξ' from an individual of a pair at distance ξ, it can destroy this pair.

There is a factor 2 before each term because the process can be applied on each individual of the pair. The pair correlation $C_{SE}(\xi, t)$ can be calculated for the SE-pairs:

$$\begin{aligned}
&\frac{dC_{SE}(\xi,t)}{dt} = \\
&Movement \begin{cases} +2\lambda \displaystyle\int \omega_1(\xi')C_{SE}(\xi+\xi')d\xi' \\ -2\lambda|\omega_1(\xi)|C_{SE}(\xi) \end{cases} \\
&Infection \begin{cases} +\beta \displaystyle\int \omega_2(\xi')T_{SSI}(\xi,\xi')d\xi' \\ -\beta \displaystyle\int \omega_2(\xi')T_{SEI}(\xi,\xi')d\xi' \end{cases} \\
&Latency \big\{ -\alpha C_{SE}(\xi)
\end{aligned} \tag{4}$$

The susceptible-exposed pair correlation dynamics depends on the three processes in the above equation:

- The movement terms (the first two) are computed in the same way as the C_{SS} pairs.
- The infection terms create and destroy some SE-pairs. The creation of a SE-pair depends on the T_{SSI} configuration and the interaction kernel ω_2. When the susceptible individual of a SS-pair is infected by an infectious individual located at distance ξ', it creates a new SE-pair. The destruction of a SE-pair depends on the T_{SEI} configuration and the interaction kernel ω_2. When the susceptible individual of a SE-pair is infected by an infectious individual located at distance ξ', it destroys the SE-pair.

- The latency term corresponds to the number of exposed individuals in a configuration at a distance of ξ with a susceptible individual and hence create a new SI-pair at a distance of ξ if they become infectious.

The dynamics of the second moments depends on the third moment $T_{ijk}(\xi, \xi')$. This moment has to be closed to perform the calculation. The moment closure is dealt with in Section B.1.

B.1 Moment closure

To achieve a closed dynamic system, the highest spatial moment must be replaced by a function of lower-order moments. This expression constitutes the moment closure. In our case, the third moment must be replaced by a function of the first and second moment. Moment closures are a key issue for moment approximation because the quality of the moment approximation is directly linked to the used moment closure. Several closures have been tested and studied in the literature [3]. The idea is to use different closures which have been tested by [3]. More details are available in these references, especially for the all properties linked to the different closures. We used power-2 closures already used by [3]. Power-2 closures are obtained by multiplying two of the three pair densities and by dividing by the density of the opposite corner. In our case, it is important to take into account the correlation that leads to the infection of susceptible individuals. This leads to the following approximation of the third moment:

$$T_{ijk}(\xi, \xi') = \frac{C_{ij}(\xi)C_{ik}(\xi')}{N_i} \tag{5}$$

If we consider this closure and the third moment used in the approximation, the correlation involved in the infection process is taken into account.

B.2 Discretization

To implement the moment approximation, it is necessary to discretize the correlation functions. For this purpose, the same discretization as in the individual-based model is used. The movement kernel and the interaction kernel are the kernels described in appendix A. An infection of susceptible occurs only if an infectious individual is located in the same cell. The individuals move to an adjacent cell with a uniform probability. The current problem is considered as bidimensional. In other terms, we consider a correlation matrix. This leads to a simplification of the dynamics as follows:

$$\frac{dN_S}{dt} = -\beta C_{SI}(0,0) \tag{6}$$

$$\frac{dN_I}{dt} = \beta C_{SI}(0,0) - \alpha N_e \tag{7}$$

$$\frac{dN_I}{dt} = \alpha N_e - \gamma N_i \tag{8}$$

for the first moment dynamics. For the second moment dynamics, we have:

$$\begin{aligned} \frac{dC_{SS}(x_i, x_j, t)}{dt} &= \\ Movement &\begin{cases} 2\lambda \sum_{i=0}^{2} \sum_{j=0}^{2} C_{SS}(x_{i-1}, x_{j-1}) \\ -\, 2\lambda C_{SS}(x_i, x_j) \end{cases} \\ Infection &\left\{ -\, 2\beta T_{SSI}([x_i, x_j], [0,0]) \right. \end{aligned} \tag{9}$$

$$
\begin{aligned}
&\frac{dC_{SI}(x_i,x_j,t)}{dt} = \\
&Movement \begin{cases} +\, 2\lambda \displaystyle\sum_{i=0}^{2}\sum_{j=0}^{2} C_{SI}(x_{i-1},x_{j-1}) \\ -\, 2\lambda C_{SI}(x_i,x_j) \end{cases} \\
&Infection \begin{cases} -\, \beta T_{SII}([x_i,x_j],[0,0]) \\ -\, \beta C_{SI}(x_i,x_j)\delta(x_i)\delta(x_j) \end{cases} \\
&Recovery \{ \; -\, \gamma C_{SI}(x_i,x_j) \\
&Latency \{ \; -\, \alpha C_{SE}(x_i,x_j)
\end{aligned} \tag{10}
$$

$$
\begin{aligned}
&\frac{dC_{SE}(x_i,x_j,t)}{dt} = \\
&Movement \begin{cases} +\, 2\lambda \displaystyle\sum_{i=0}^{2}\sum_{j=0}^{2} C_{SE}(x_{i-1},x_{j-1}) \\ -\, 2\lambda C_{SE}(x_i,x_j) \end{cases} \\
&Infection \begin{cases} +\, \beta T_{SSI}([x_i,x_j],[0,0]) \\ -\, \beta T_{SEI}([x_i,x_j],[0,0]) \end{cases} \\
&Latency \{ \; -\, \alpha C_{SE}(x_i,x_j)
\end{aligned} \tag{11}
$$

B.3 Computer implementation

The MA model was implemented with Matlab software (using Mathworks ©).

References

1. Quesnel G, Duboz R, Ramat É (2009) The Virtual Laboratory Environment – An operational framework for multi-modelling, simulation and analysis of complex dynamical systems. Simulation Modelling Practice and Theory 17: 641–653.

2. R Development Core Team (2006) R: A Language and Environment for Statistical Computing. R Foundation for Statistical Computing, Vienna, Austria. ISBN 3-900051-07-0.

3. Dieckmann U, Law R (2000) The geometry of ecological interactions: simplifying spatial complexity. Cambridge, UK: Cambridge University Press.
